# Treatment of severe borderline personality disorder with clozapine

**DOI:** 10.4103/0019-5545.70989

**Published:** 2010

**Authors:** Adarsh Kumar Vohra

**Affiliations:** Consultant Psychiatrist and College Tutor Parkwood, East Park Drive Blackpool, FY3 8PW, Lancashire, United Kingdom

**Keywords:** Case report, clozapine, borderline personality disorder, severe

## Abstract

Patients with borderline personality disorder (BPD) show significant impairment in the domain of interpersonal and social functioning and may use the resources of health and social services extensively, with little beneficial outcome. At present there are no clear guidelines in literature for the use of pharmacotherapy in the management of BPD. According to the scanty literature available in the form of case reports and small studies, clozapine has been demonstrated to be effective in the management of BPD. The case presented here describes the effectiveness of clozapine in a young female patient with severe BPD (without psychotic features), who had repeated and prolonged periods of hospitalization and was successfully treated with a moderate dose of clozapine, following a failure to improve with other psychotropic medications. More studies are suggested to evaluate the efficacy of clozapine in patients with BPD, both with and without psychotic features, to find out the optimum dose and to weigh the risk and benefits of clozapine.

## INTRODUCTION

According to DSM-IV-TR the diagnosis of borderline personality disorder (BPD) requires five out of nine specific diagnostic criteria. These nine specific diagnostic criteria are: fear of abandonment, unstable and intense relationship, identity disturbances, impulsivity, recurrent suicidal behavior, affective instability, chronic feeling of emptiness, inappropriate intense anger, and transient stress-related paranoid ideation or severe dissociative symptoms.[1] The prevalence of BPD is about 2% and 20–40% in the general population and among psychiatric patients, respectively.[2] Patients with severe BPD show significant impairment in both interpersonal and social functioning and can extensively use the resources of health and social services, with little benefit.[3]

At present there are no clear guidelines in the literature for the pharmacological treatment of BPD.[4] Clozapine appears to be the most effective anti-psychotic, but has limited use because of its risk of causing agranulocytosis in 1% of the patients.[5] Clozapine is reported to be effective in the management of BPD, lowering the risk of suicide,[6] and in decreasing the severity of aggressive symptoms.[7] The therapeutic use of clozapine in BPD has not been studied widely, but according to case reports and small studies, clozapine has been shown to be useful in the management of cases of BPD, in terms of reducing self-harm, aggression, and other associated symptoms.[8–10] The beneficial effects of clozapine have also been noticed in the reduction of hostility[7] and self-mutilation.[10] Because of the broad spectrum of neuroreceptor pharmacology, there are negligible extrapyramidal side effects and significant improvement in some patients; the use of clozapine has led to its off-label use in certain psychiatric conditions, such as, bipolar psychoses, L-dopa-induced psychoses, and psychoses associated with dementias.[11]

This report looks at a young female patient with a diagnosis of BPD who had repeated and prolonged hospitalization and was successfully treated with clozapine following failure to improve with other psychotropic medications.

## CASE REPORT

Ms. X is a 24-year-old, single, white woman who was admitted following an overdose of zopiclone and lorazepam soon after she took her own discharge from the hospital. At the time of admission she was agitated, distressed, and challenging in her behavior. She displayed no remorse following her overdose and expressed plans to further harm herself.

Ms. X had her first contact with the psychiatric services at the age of 10 and was diagnosed with conduct disorder. Since then, she harmed herself on numerous occasions, remained in regular contact with psychiatric services as an outpatient and an inpatient, and spent periods of time in the psychiatric intensive care unit (PICU). She started abusing Cannabis around the age of 11 and thereafter abused amphetamines, ecstasy, heroin, solvents, and alcohol. She was arrested for petty theft and breach of peace on few occasions. Ms. X is the youngest of four siblings. Her mother suffered from mental health problems that resulted in all her four children, including Ms X, going into foster care. Ms. X often used to run away from home and steal on a regular basis, and was referred to the community adolescent mental health services team at the age of 14.

She moved into a series of temporary accommodations following discharge from care, at the age of 16. Around this time she took an overdose for the first time and was admitted to a hospital where she was prescribed antidepressants. From this point on she began to manage her feelings and emotions of emptiness, boredom, and frustration through self-harm. Over the years she continued to harm herself on a regular basis and had several long and protracted stays as an inpatient. While in the community she consistently placed herself in situations that left her vulnerable to danger. Her behavior ranged from taking drugs and mixing with other high-risk users, displaying aggressive and impulsive behavior, such as walking out onto the roads, standing on the edge of bridges, and taking numerous overdoses. During this long period of contact with the psychiatric services, under a well-qualified team of psychiatrists, nurses, psychologists, and social workers, she never qualified for the diagnosis of depressive disorder as a clinical entity. However, she did present few fluctuating depressive symptoms and mood instability over a long period of time, but never presented with psychotic symptoms.

During this entire period she received intensive psychological input, including psychotherapy, and a series of different psychotropic medications, including antidepressants and anti-psychotics, at different periods of time in a considerable dose and for an adequate period of time. Unfortunately she failed to show any consistent or appreciable improvement.

During her last admission she showed very challenging behavior, was often verbally and physically abusive toward the staff, would indulge in self-harm in the form of inflicting injuries on herself, cutting herself, and tying a ligature around her neck. She was placed in restraint on numerous occasions. In the past she had been treated with different antidepressants and anti-psychotic medications. Finally three months after her current admission she consented to take clozapine that was gradually titrated from a small dose of 12.5 mg to 175 mg daily as per the Maudsley prescribing guidelines, for a period of three weeks. Following registration with the Clozaril Patient Monitoring *Service* (CPMS), for clozapine administration, in the UK, she was monitored for daily pulse, blood pressure, and mandatory blood testing for white cell count (WCC) and differential blood count on a weekly basis for the first 18 weeks, and then every two weeks for the remainder of the year, and thereafter on a monthly basis. She tolerated clozapine treatment reasonably well, apart from weight gain and transit tachycardia. After 8–10 weeks of the commencement of clozapine she started showing improvement, her mood improved, she felt far happier and her self-harming behavior dramatically decreased in terms of frequency and severity. Unfortunately she gained weight as a side effect of clozapine, but she stated, “rather be fat and happy, than skinny and miserable”. She improved to such a degree that she felt “totally in control of her life”. She expressed no thoughts of self-harm and never had any evidence of psychotic features. She could have a good relationship with her care co-ordinator and made several friends in the community on whom she could rely for company and support. She was discharged after one year of admission in the psychiatric ward.

## DISCUSSION

At present there are no clear guidelines for psychotropic drug use in BPD. Serotonin specific reuptake inhibitors (SSRIs) are indicated to control impulsive behavior and comorbid affective disorder.[9] Benzodiazepines, mood stabilizers, and neuroleptics may be indicated in controlling aggression and psychotic symptoms in BPD.[9,10] Clozapine has been shown to be effective in controlling aggressive behavior, irrespective of the underlying pathology, such as in autism and BPD.[9,11] The improvement of aggression in patients with schizophrenia receiving clozapine has been an interesting observation. The issue of whether clozapine’s efficacy is due to a specific anti-aggressive effect or is merely a reflection of an overall improvement in psychosis has been discussed, and it has been suggested that clozapine has a distinct anti-aggressive effect,[7] which is not linked with its sedative properties.[12] Clozapine has also been reported to be effective in reducing hostility besides being useful in the improvement of psychosis[7] and suicide in schizophrenia.[13] The improvement exhibited in the patient presented by the author could be seen in line with the views expressed by other authors of similar studies stating that clozapine reduces aggressive behavior, hostility, and suicidal risk.

There are only few studies conducted to detect the effectiveness of clozapine in BPD patients. A study on 15 BPD patients treated with clozapine showed improvement in psychotic symptoms and overall global functioning.[14] In 1998, another study on 12 BPD patients treated with a low dose of clozapine (25–100 mg per day) confirmed improvement on the Brief Psychiatric Rating Scale (BPRS) and Global Assessment of Functioning (GAF) score, impulsivity, and mood.[9] Similarly Swinton[15] has shown clozapine to be effective in reducing symptoms of psychosis and levels of self-injury in BPD. The patient presented here failed to demonstrate improvement with conventional pharmacological treatment recommended for the treatment of BPD, but showed significant improvement in impulsive, aggressive, and self-injurious behavior, with a dose of 175 mg/day of clozapine, leading to improvement in overall functioning. This clearly lends support to the findings reported in various studies on the effectiveness of clozapine in BPD patients who fail to respond to conventional pharmacotherapy.[8,9,14] The presentation of this patient is also in line with the findings of Swinton,[15] who also demonstrated that a group of patients who were regarded as untreatable and difficult to manage showed considerable improvement with clozapine.

The unusual feature of the study conducted by Benedetti *et al*.[9] was the use of a fairly small dose of clozapine (average 44 mg/day: range 25–100 mg/day). Another study reported a reduction in the episodes of self-injury and assaults, and a need for seclusion, in seven long-term inpatients of BPD, treated with conventional doses of clozapine.[8] However, the patient reported by the author, improved with a moderate dose of clozapine (175 mg/day).

Unlike a majority of the patients of BPD, as reported in the literature, this patient did not manifest any psychotic symptoms, but improved considerably with clozapine. This finding is consistent with the results of the study carried out by Parker,[16] in which clozapine is effective in severe cases of BPD even in the absence of psychotic features.

The nontrivial risk of agranulocytosis, other side effects, and cost related to clozapine, do pose a limitation to its broad use for the patients of BPD and warrants further studies.[17] Similarly further studies are required to evaluate the effectiveness of clozapine in patients with BPD, both with and without psychotic features, to identify the optimum and effective dose, and to weigh the risk and benefits of clozapine.[18]
